# Complete chloroplast genome of an endangered tree species, *Toona ciliata* (Sapindales: Meliaceae)

**DOI:** 10.1080/23802359.2018.1476074

**Published:** 2018-06-08

**Authors:** Gui-Liang Xin, Jia-qian Liu, Jia Liu

**Affiliations:** Key Laboratory of Resource Biology and Biotechnology in Western China (Northwest University), Ministry of Education, School of Life Science, Northwest University, Xi’an, China

**Keywords:** *Toona ciliate*, chloroplast genome, endangered species, phylogenetic analysis

## Abstract

The whole cp genome of *T. ciliata* was 159,502 bp in length, containing a pair of inverted repeat (26,961 bp for each), a large single copy (87,199 bp) and a small single copy (18,381 bp) regions. The cp genome encoded 138 genes, including 89 protein-coding genes, 40 tRNA genes, 8 rRNA genes and 1 pseudogene. The nucleotide composition was asymmetric (30.7% A, 19.3% C, 18.6% G and 31.4% T) with an overall GC content of 37.9%. The maximum likelihood phylogenetic analysis based on 21 complete cp genome sequences showed that *T. ciliata* closely related to Azadirachta indica.

*Toona ciliate* is endemic to tropical and subtropical China. High value and huge potential use of *T. ciliate* directly incurred human overcutting and consequent habitat destruction during the last century, and thus, International Union for Conservation of Nature (IUCN) has included it in the Red List of Endangered species (Version 2.3, 2017). Some measurements have been taken to conserve this important species since 1991 in China (Zhao et al. 2015; Zhou et al. 2015). However, much still remained unknown about the complete chloroplast (cp) genome. In this study, we assembled the complete cp genome of *T. ciliata* based on the whole-genome Illumina sequencing dataset to improve an appreciation of its genetics that would be conducive to the formulation of conservation and management strategies.

Young leaves were collected from a single individual of *T. ciliata* at the campus of Fujian Agriculture and Forestry University (Fujian, China; 26°03′N, 119°16′E), and were dried using silica gel. Then, total genomic DNA was extracted using modified CTAB protocol (Yang et al. 2014) for the construction of a shotgun library, which was sequenced using the Illumina Hiseq 2000 Platform (Illumina, San Diego, CA). In total, 27,617,866 of 150 bp raw paired-end reads were obtained and the cp genome was assembled by MITObim v1.8 (Hahn et al. 2013) with the sequences of published *Azadirachta indica* (KF986530) cp genomes as the initial reference.

The cp genome of *T. ciliata* was annotated using software Geneious v 9.0.2 (Biomatters Ltd., Auckland, New Zealand) via comparison with the cp genome of relatively related species, including *A. indica* (KF986530), *Leitneria floridiana* (KT692940), and *Litchi chinensis* (KY635881). Finally, the physical map of the circular cp genome was generated using OGDRAW (Lohse et al. 2013).

The whole cp genome was 159,502 bp in length, containing a pair of inverted repeat (26,961 bp for each), a large single copy (87,199 bp) and a small single copy (18,381 bp) regions. The cp genome encoded 138 genes, including 89 protein-coding genes (77 unique), 40 tRNA genes (29 unique), eight rRNA genes (four unique), and one pseudogene. The nucleotide composition was asymmetric (30.7% A, 19.3% C, 18.6% G, and 31.4% T) with an overall GC content of 37.9%.

To investigate phylogenetic status of *T*. *ciliate*, a maximum likelihood (ML) analysis was reconstructed from the complete cp genomes of 19 species by the RAxML software (Stamatakis 2014) performed with 1000 replicates (Figure 1). The phylogenetic analysis indicated that *T. ciliata* was closely related to *A. indica*. The complete cp genome of *T. ciliata* will supply useful genetic information for population genomic studies, and conservation management of this endangered species.

**Figure 1.**
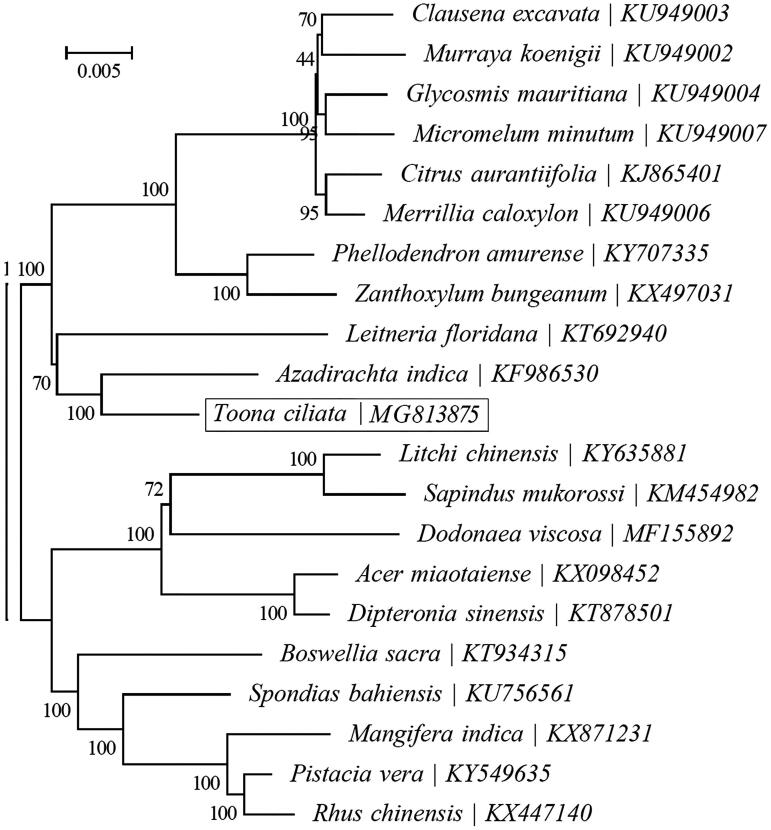
ML phylogenetic tree based on 21 complete cp genome sequences. The bootstrap values are indicated next to the branches.
